# RNA Modifications and Epigenetics in Modulation of Lung Cancer and Pulmonary Diseases

**DOI:** 10.3390/ijms221910592

**Published:** 2021-09-30

**Authors:** Pai-Chi Teng, Yanwen Liang, Aliaksandr A. Yarmishyn, Yu-Jer Hsiao, Ting-Yi Lin, Tzu-Wei Lin, Yuan-Chi Teng, Yi-Ping Yang, Mong-Lien Wang, Chian-Shiu Chien, Yung-Hung Luo, Yuh-Min Chen, Po-Kuei Hsu, Shih-Hwa Chiou, Yueh Chien

**Affiliations:** 1 Department of Medical Research, Taipei Veterans General Hospital, Taipei 112027, Taiwan; paichi.teng@gmail.com (P.-C.T.); yanwen95ktb@gmail.com (Y.L.); yarmishyn@gmail.com (A.A.Y.); yj1007hsiao@yahoo.com (Y.-J.H.); lintingyi2014@gmail.com (T.-Y.L.); backyard0826@gmail.com (T.-W.L.); andrea_chi@hotmail.com (Y.-C.T.); molly0103@gmail.com (Y.-P.Y.); monglien@gmail.com (M.-L.W.); cschien6688@gmail.com (C.-S.C.); 2 Department of Life Sciences, Institute of Genomic Sciences, National Yang Ming Chiao Tung University, Taipei 112304, Taiwan; 3 School of Medicine, National Yang Ming Chiao Tung University, Taipei 112304, Taiwan; 4 Institute of Pharmacology, National Yang Ming Chiao Tung University, Taipei 112304, Taiwan; 5 Department of Ophthalmology, Taipei Veterans General Hospital, Taipei 112201, Taiwan; 6 Institute of Food Safety and Health Risk Assessment, National Yang-Ming Chiao Tung University, Taipei 112304, Taiwan; 7 Genomic Research Center, Academia Sinica, Taipei 11529, Taiwan; 8 Department of Bioengineering, Institute of Engineering in Medicine, University of California San Diego, La Jolla, CA 92093, USA; 9 Department of Chest Medicine, Taipei Veterans General Hospital, Taipei 112201, Taiwan; hecterlo@gmail.com (Y.-H.L.); ymchen@vghtpe.gov.tw (Y.-M.C.); 10 Division of Thoracic Surgery, Department of Surgery, Taipei Veterans General Hospital, Taipei 112201, Taiwan; hsupokuei@yahoo.com.tw

**Keywords:** RNA epigenetics, lung cancer, methylation, N6-methyladenosine, pulmonary diseases

## Abstract

Lung cancer is the leading cause of cancer-related mortality worldwide, and its tumorigenesis involves the accumulation of genetic and epigenetic events in the respiratory epithelium. Epigenetic modifications, such as DNA methylation, RNA modification, and histone modifications, have been widely reported to play an important role in lung cancer development and in other pulmonary diseases. Whereas the functionality of DNA and chromatin modifications referred to as epigenetics is widely characterized, various modifications of RNA nucleotides have recently come into prominence as functionally important. N6-methyladosine (m6A) is the most prevalent internal modification in mRNAs, and its machinery of writers, erasers, and readers is well-characterized. However, several other nucleotide modifications of mRNAs and various noncoding RNAs have also been shown to play an important role in the regulation of biological processes and pathology. Such epitranscriptomic modifications play an important role in regulating various aspects of RNA metabolism, including transcription, translation, splicing, and stability. The dysregulation of epitranscriptomic machinery has been implicated in the pathological processes associated with carcinogenesis including uncontrolled cell proliferation, migration, invasion, and epithelial-mesenchymal transition. In recent years, with the advancement of RNA sequencing technology, high-resolution maps of different modifications in various tissues, organs, or disease models are being constantly reported at a dramatic speed. This facilitates further understanding of the relationship between disease development and epitranscriptomics, shedding light on new therapeutic possibilities. In this review, we summarize the basic information on RNA modifications, including m6A, m1A, m5C, m7G, pseudouridine, and A-to-I editing. We then demonstrate their relation to different kinds of lung diseases, especially lung cancer. By comparing the different roles RNA modifications play in the development processes of different diseases, this review may provide some new insights and offer a better understanding of RNA epigenetics and its involvement in pulmonary diseases.

## 1. Introduction to Lung Cancer

As of 2020, lung cancer is the second most commonly diagnosed malignancy, accounting for 11.4% of total new cases and is the leading cause of cancer-related mortality, with an estimated 1.8 million deaths (18%) worldwide [1]. Histologically, lung cancer is categorized into small-cell lung carcinoma (SCLC) and non-small-cell lung carcinoma (NSCLC). NSCLC accounts for 85% of all lung cancer cases and is further subclassified into lung adenocarcinoma (LUAD), squamous-cell carcinoma (LUSC), and large-cell carcinoma (LCC). While these histological types represent distinct diseases, they do share some common molecular attributes and risk factors. Smoking remains tightly linked to lung cancer, especially SCLC and LUSC. Yet, only about 10% of smokers ultimately develop lung cancer [2] and an estimated 15% of men and 53% of women with lung cancer are non-smokers [3]. Other risk factors of lung cancer include asbestos and environmental insults. Despite the recent intensified and rapid developments in diagnostic methods and targeted drugs [4], not all lung cancer patients benefit from effective targeted therapy, as drug resistance often hinders treatment efficacy. Failure of early diagnosis often leads to low treatment efficiency and poor prognosis as RNA epigenetic-driven developments unfold. Identifying new biomarkers and therapeutic targets with an improved understanding of the underlying molecular RNA epigenetic mechanisms is important for the early diagnosis, prognosis, and treatment of lung cancer. 

Epigenetic variability, including RNA methylation, contributes to the phenotypic makeup and risk of carcinogenesis. Presently, the use of long-term therapeutic drugs, such as tyrosine kinase inhibitors (TKIs) [5], usually leads to acquired drug resistance and henceforth disease progression in cancer patients. Studies of the recent decade have shown that this complexity in cancer development is driven by the accumulation of genetic and epigenetic alterations. Some cancer cells have also shown epigenetic and signalling pathway similarities with stem cells, recapitulating a “stem-like” state [6]. Although somatic genetic aberrations, such as mutations and copy number variations, play a crucial role in oncogenesis, epigenetic alterations arise more frequently and significantly than somatic mutations [7]. In particular, lung cancer is characterized by an accumulation of epigenetic alterations in the respiratory epithelium [8], resulting in oncogenes’ expression and the repression of tumour suppressor genes. The possibility of reversing epigenetic events may serve as a new strategy for therapeutic intervention. In this review, we discuss the dysregulation of expression of m6A-, m1A- and m7G-related proteins (i.e., their writers, readers, and erasers) and their increasing associations with the tumorigenic development of different types and subtypes of lung cancer and pulmonary diseases. These regulatory proteins are of great potential to serve as novel diagnostic biomarkers and therapeutic targets for the early diagnosis and improved prognosis and treatment of lung cancer and pulmonary diseases.

## 2. RNA Modifications

More than 170 different types of RNA modifications have been reported to date to modify the entire range of RNA types, including messenger RNAs (mRNAs), transfer RNAs (tRNAs), ribosomal RNAs (rRNAs) and long noncoding RNAs (lncRNAs) [9]. Similarly to epigenetic modifications in DNA and histones, such RNA marks can be deposited, erased and recognized by a dedicated machinery of proteins, i.e., writers, erasers and readers, respectively. Whereas N6-methyladenosine (m6A) is the most prevalent internal modification in mRNA, there are several other important modifications in mRNAs and noncoding RNAs (ncRNAs) including N1-methyladenosine (m1A), 5-methylcytosine (m5C), 7-methylguanosine (m7G), pseudouridine (Ψ, psi) and adenosine-to-inosine (A-to-I) (Table 1).

### 2.1. m6A

m6A is the most prevalent and abundant internal mRNA modification in eukaryotic cells [10,11] that influences a wide variety of RNA processing steps, including splicing, mRNA stability, and translation [12,13,14].

#### 2.1.1. m6A Writers

The m6A marks are deposited co-transcriptionally by the multicomponent m6A methyltransferase complex, which mainly consists of METTL3/METTL14 heterodimer at its core [15,16], and various auxiliary subunits including WTAP [17], RBM15 [18], RBM15B [18], and VIRMA [19,20]. METTL3 possesses the methyltransferase activity that transfers the methyl group from S-adenosyl methionine (SAM). In addition, METTL16 has also been reported to function as an m6A methyltransferase, but only methylating a limited number of RNAs with specific RNA structures (e.g., U6 snRNA).

##### METTL3

Methyltransferase-like 3 (METTL3) is a member of the SAM-dependent methyltransferase family. It is a highly conserved protein with homologs in yeast, plants, *Drosophila*, and mammals. METTL3 recognizes the sequence motif GGACU and adds a methyl group to its adenosine residue using SAM as a methyl group donor. It is mostly localized to the nucleus with particular enrichment in the nuclear speckles [17]. METTL3 was also shown to localize to the cytoplasm, where it could perform the methyltransferase-independent function in the initiation of translation [21]. The deletion of METTL3 in mammalian cells results in a huge loss of m6A in the mature mRNA, while its knockout in mice leads to embryonic lethality [22]. Thus, METTL3 is involved in a plethora of biological processes including embryonic development, cell reprogramming, and spermatogenesis [23].

##### METTL14

METTL14 is the major supporting subunit of the m6A methyltransferase complex that strengthens the catalytic effect of m6A RNA methylation. Although METTL14 is also a member of the METTL family of enzymes, its SAM-binding domain is not functional. Therefore, it lacks the enzymatic activity to generate m6A. On the other hand, METTL14 serves as an RNA-binding scaffold that facilitates substrate recognition [24]. 

##### WTAP

Wilms Tumor 1-Associating Protein (WTAP) is a regulatory subunit of the METTL3/METTL14 methyltransferase complex [25]. Whereas WTAP lacks methyltransferase domain and associated catalytic activity and does not have any effect on the activity of the METTL3/METTL14 in vitro, its main function in vivo was shown to be the regulation of recruitment of the METTL3/METTL14 complex to the nuclear speckles, the regions dedicated to transcription and pre-mRNA processing [16,26]. The knockdown of WTAP demonstrated even more drastic effects on the m6A levels then the depletion of METTL3 or METTL14, indicative of its crucial functions [19,27].

##### VIRMA (KIAA1429)

Vir-like m6A methyltransferase associated (VIRMA) is another component of the m6A methyltransferase complex. The knockdown of VIRMA in lung cancer cells resulted in a four-fold depletion of the m6A level, indicating its important function in the m6A methyltransferase complex [19]. In addition, VIRMA was shown to selectively mediate m6A mRNA methylation in the 3′-UTR and close to stop codons, which was associated with alternative polyadenylation [28]. 

##### RBM15/RBM15B and ZC3H13

RBM15 and its paralog, RBM15B, were first identified through the proteomic analysis of proteins binding to WTAP [18,29]. It was reported that RBM15 and RBM15B could interact with METTL3 through WTAP [18]. The knockdown of RBM15 and RBM15B led to the depletion of m6A levels, indicative of their significant role in m6A deposition. The iCLIP studies have shown that RBM15 and RBM15B bind to the U-rich regions in mRNA proximal to existing m6A modifications [18]. Thus, RBM15 and RBM15B may function as recruiters which guide the WTAP/METTL3 catalytic complex to already existing m6A sites to generate new m6A marks in their vicinity. Recently, it was revealed that Flacc, the *Drosophila* homolog of the mammalian ZC3H13, serves as an adaptor between Nito (RBM15B) and Fl(2)d (WTAP), which resulted in the stabilization of the complex and the elevation of the deposition of m6A on mRNA [30]. 

##### METTL16

METTL16 is a paralog of METTL3, whose role of a new *bona fide* m6A writer was recently confirmed [31]. METTL16 was found to methylate the hairpin structures in U6 small nuclear RNA and *MAT2A* pre-mRNA, which encodes an enzyme involved in the biosynthesis of SAM [31]. In addition, METTL16 was also reported to associate with various other pre-mRNAs and ncRNAs [32]. Compared to METTL3, clearly distinct structural features, such as a large positively charged groove responsible for RNA binding, were found in the 3D structure of METTL16 [33].

#### 2.1.2. m6A Readers

The consequences of m6A modifications of mRNAs are dependent on the direct recruitment of the RNA binding proteins referred to as m6A readers. The most characterized m6A readers belong to the YT521-B homology (YTH) domain family, originally identified in an m6A pull-down assay [34]. In addition, several other proteins were reported to bind to m6A, including IGF2BP1-3. The binding of different m6A readers directs mRNAs into specific pathways of RNA metabolism such as splicing, decay, translation, and localization. 

##### YTH Domain Proteins

The YTH domain binds to m6A-methylated mRNA with 10–50 times higher affinity than to unmethylated mRNA [35,36,37]. Mammals have five members of the YTH domain family m6A readers, which are subcategorized into two subfamilies: DF (YTHDF1-3) and DC (YTHDC1-2).

YTHDF1 was originally identified to bind to m6A marks located close to stop codons and 3′-UTRs, and its overall distribution pattern was highly consistent with the m6A site distribution. YTHDF1 was demonstrated to directly bind to the eukaryotic translation initiation factor eIF3, thus enhancing the translation efficiency of m6A-modified RNA targets [38]. 

The most defined function of YTHDF2 is mediation of the degradation of m6A-modified transcripts. YTHDF2 normally co-localizes with the deadenylase complex and decapping complex proteins and recruits its target mRNAs into the P-bodies for degradation [36]. Subsequent study showed that YTHDF2 accelerates the degradation of m6A-modified transcripts by recruiting CCR4-NOT deadenylation complex [39].

As was demonstrated by the PAR-CLIP-Seq analysis, YTHDF3 and YTHDF1 bind to the similar RNA motifs, and the binding sites are predominantly localized to the 3′-UTRs. YTHDF3 can promote the translation efficiency of consensus target genes of YTHDF1, indicative of synergistic regulation of translation efficiency by these two m6A readers. Moreover, YTHDF3 can also mediate mRNA degradation by directly interacting with YTHDF2 [14,40]. 

Unlike cytoplasmic DF m6A readers, YTHDC1 is localized to the nucleus. It has been shown to interact with the serine-arginine repeat (SR) splicing factors such as SRSF3 and SRSF10, in such a way regulating m6A-mediated exon inclusion type alternative splicing [41]. Moreover, YTHDC1 was also demonstrated to interact with m6A marks on *XIST* mRNA and promote its function in X chromosome inactivation [18].

YTHDC2 is the largest member of the YTH family, and it tends to bind to the conserved m6A-modified motifs. YTHDC2 regulates the stability of m6A-edited mRNAs and interacts with both m6A-modified mRNAs and the ribosomes to facilitate translation efficiency [25,26]. YTHDC2 was shown to play a particularly important role in mammalian spermatogenesis [27,42].

##### IGF2BPs

Insulin-like growth factor 2 mRNA-binding proteins (IGF2BPs; including IGF2BP1/2/3) are mRNA-binding proteins that are highly conserved from fish to humans [43] and were characterized as important regulators of translation, stability, splicing and intracellular localization of targeted RNAs [44]. Recently, IGF2BPs were found to associate with m6A by RNA via their K homology (KH) domains and promote oncogenesis by stabilizing oncogenic mRNAs such as *MYC* [45].

#### 2.1.3. m6A Erasers

Unlike the m6A methyltransferase, which requires a protein complex composed of various regulatory subunits for its catalytic ability, m6A demethylases (m6A erasers) act as single proteins. Currently, two mammalian m6A demethylases are known, fat mass and obesity-associated protein (FTO) and alpha-ketoglutarate-dependent dioxygenase alkB homolog 5 (ALKBH5).

##### FTO

Fat mass and obesity-associated protein (FTO) was the first identified mammalian RNA m6A demethylase [46]. The knockdown of FTO resulted in a substantial increase of m6A in mRNA [46]. In addition to removing m6A marks, FTO was also reported as the major demethylase to catalyse the removal of the m6Am modification occurring next to the mRNA 5′-caps [47]. Although the major biological function FTO was first identified to be associated with obesity [48], recently it was also characterized as a potent oncogene in leukaemia [49] and glioblastoma [50]. 

##### ALKBH5

Alpha-ketoglutarate-dependent dioxygenase AlkB homolog 5 (ALKBH5) is another known m6A demethylase. It has been reported that the high expression of ALKBH5 in mouse testis is essential for spermatogenesis and thus mouse reproduction. The level of mRNA m6A is elevated in the male mice with ALKBH5 deficiency, which results in impaired fertility due to the apoptosis of spermatocytes in the metaphase of meiosis [51].

### 2.2. N1-Methyladenosine (m1A)

N1-methyladenosine (m1A) is another type of RNA methylation mark of moderate abundance that was found to modify tRNAs, rRNAs and mRNAs. Human mitochondrial tRNAs are known to contain m1A marks at positions 9 (m1A9) and 58 (m1A58), which are catalysed by TRMT10C and TRMT61B methyltransferases, respectively [52]. In the vertebrate mitochondrial ribosome, m1A is conserved in the position 947 of the 16S rRNA, which is likely to stabilize it [53]. In the nuclear-coded large rRNA, specific m1A site located in the peptidyl transfer centre of the ribosome is conserved between budding yeast and humans [32,54,55]. In addition, m1A is also found at position 1322 of 28S rRNA, which is catalysed by the human nucleolar protein nucleomethylin (NML; also known as RRP8) [52]. m1A58 is normally erased by ALKBH1 [52] and m1A marks in mRNAs are erased by ALKBH3 demethylases [56]. FTO was demonstrated to directly repress translation by catalysing m^1^A tRNA demethylation [10.1016/j.molcel.2018.08.011]. It was found that several YTH family proteins, such YTHDF1-3 and YTHDC1, but not YTHDC2, could bind to m1A marks, thus serving as potential readers [57]. 

### 2.3. m5C

RNA 5-methylcytosine (m5C) exists on rRNAs, tRNAs, mRNAs, ncRNAs and enhancer RNAs (eRNAs) [58]. m5C is associated with different functions in different RNA subtypes. For example, in tRNAs, m5C is related to conferring RNA structure and stability, and is also required for translation accuracy [59], while in 25S rRNA, the loss of modification at position 2278 causes the translational readthrough of stop codons [60].

NSUN1 to NSUN7, and DNA methyltransferase-like 2 (DNMT2) were reported as m5C writers. NSUN1 and NSUN5 modify 28S rRNA, while NSUN3 and NSUN4 modify mitochondrial tRNA and rRNA, respectively. As for NSUN2 and DNMT2, they modify cytoplasmic tRNA, while NSUN7 targets eRNAs as a substrate. Additionally, NSUN2 can also modify the noncoding Vault RNA and mRNAs [58].

### 2.4. m7G

7-Methylguanosine (m7G) was first identified at the initial positions in mRNAs [61], and internally in rRNAs [62] and tRNAs [63]. Recently, it was detected internally in human mature miRNAs and miRNA precursors [64]. m7G modification on tRNAs is mediated by the METTL1–WDR4 complex [65], and on rRNA is mediated by WBSCR22 [66].

### 2.5. Pseudouridine (Ψ, psi)

Pseudouridylation is the most abundant modification found in total RNA from human cells. It is particularly abundant in rRNA and is necessary for ribosome assembly [67]. Pseudouridylation of U RNAs, a specific subtype of snRNAs which play an important role in splicing, is necessary for efficient mRNA splicing, especially under the pressure of cellular stress [68]. 

The effect of pseudouridylation on mRNA still needs further study. Pseudouridine (Ψ) appears to be enriched within the coding regions and 3′-UTRs of mRNA, but it is not associated with any specific features within these regions [69]. No sequence motif has been associated with Ψ marks, but a specific RNA structural signature seems to be necessary and sufficient for pseudouridylation of mRNA [70].

### 2.6. Adenosine-to-Inosine (A-to-I) Editing

While another kind of RNA-editing, cytosine-to-uridine conversion, was shown to mediate an isoform shift in the apolipoprotein B (*APOB*) transcript by introducing an early stop codon [71,72]. The APOBEC cytidine deaminase family mediates adenosine-to-inosine editing of the RNA editing events in vertebrates belong to the second category, adenosine-to-inosine (A-to-I) conversion, mediated by adenosine deaminase acting on dsRNA 1 (ADAR1) and ADAR2. In contrast, a third family member, ADAR3, seems to lack enzyme activity and may function as a dominant negative [73]. Adenosine deaminase acting on tRNA 2 and 3 (ADAT2 and ADAT3), two additional enzymes work as a complex to edit A34 in specific tRNAs [73]. As inosines preferentially pair with cytidines rather than with uridines, therefore they are read as guanosines by the ribosome, A-to-I conversion can modify the amino acid sequence of proteins, modify the secondary structure of RNA, regulate splicing and change the target specificity of miRNAs. This type of editing is mainly found on mRNA primary transcripts, tRNAs and miRNAs [73].

## 3. RNA Modifications in Lung Cancer

### 3.1. m6A Modifications in Lung Cancer

m6A is the most prevalent and abundant internal mRNA modification in eukaryotic cells [10,11] which modulates a wide variety of RNA processing steps, including splicing, mRNA stability, and translation [12,13,14]. m6A has also been found to upregulate glycolysis in hepatocellular carcinoma and colorectal carcinoma [74]. Recent studies have revealed the critical epitranscriptomic roles of dysregulated m6A modifications in lung cancer [13,21,75,76]. Thus far, different studies have demonstrated that the m6A modification of mRNA regulates self-renewal and the cell fate of cancer cells [77]. The aberrant expression of m6A RNA methyltransferases and demethylases was associated with the dysfunction of RNA-dependent functions in lung cancer. These reversible m6A marks on the transcripts can function as novel biomarkers, demonstrating the increasing prospects for lung cancer’s early diagnostics and therapeutics.

#### 3.1.1. Involvement of m6A Writers in Lung Cancer

Several recent studies have highlighted the role of m6A writer proteins in regulating lung cancer stem cells [76]. For one, the oncogenic roles of the m6A writer protein METTL3 have been reported in NSCLC. METTL3 was observed to be overexpressed in human lung cancer tissues compared with normal tissues [9,78]. The elevated levels of METTL3 were shown to promote tumour growth by catalysing m6A methylation of not only mRNAs, but also noncoding RNAs, correlating with tumour stages in primary lung adenocarcinoma samples [21,79]. The knockdown of METTL3 resulted in increased cell apoptosis and modulation of p53 signalling in cancer cells, suggesting that it might be essential for cancer cell survival [80,81].

METTL3 is the main m6A writer protein which serves as the catalytic subunit of the complex responsible for m6A modifications. Previous studies have revealed METTL3 activity in both the nucleus and the cytoplasm [82]. Lin et al. (2016) found that cytoplasmic METTL3 can directly promote the translation of several pro-tumorigenic mRNAs, including the epidermal growth factor receptor (*EGFR*) and the Hippo pathway effector *TAFAZZIN*, by recruiting the eukaryotic initiation factor 3 (eIF3). This oncogenic role of METTL3 relies on its translation-promoting ability, which is surprisingly independent of its methyltransferase catalytic activity [21].

**Figure 1:**
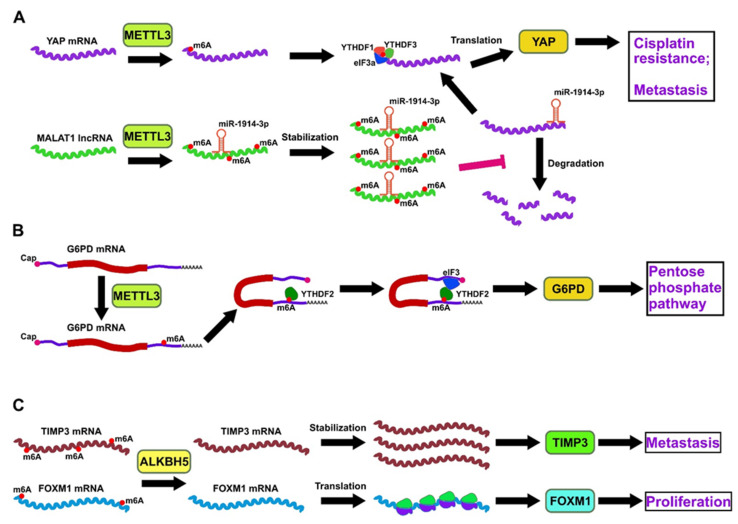
Some representative pathways of the involvement of m6A writers, readers and erasers in lung cancer. (**A**) METTL3 regulates YAP oncogene via direct m6A modification of its 5′-end allowing its YTHDF1/3-dependent enhancement of translation, and via indirect way by m6A-mediated stabilization of *MALAT1* lncRNA, which in its turn sponges YAP-targeting miR-1914-3p. (**B**) METTL3-dependent deposition of m6A at 3′-UTR of G6PD-encoding mRNA enhances its translation via YTHDF2-dependent eIF2 recruitment leading to pro-oncogenic activation of the pentose phosphate pathway. (**C**) ALKBH5 demethylates *TIMP3* and *FOXM1* oncogenic mRNAs, causing increased stability of the former and increased translation of the latter.

METTL3 has been shown to be involved in the dissemination and metastasis of lung cancer cells by mediating the epithelial-mesenchymal transition (EMT) by a mechanism dependent on its m6A catalytic activity [83]. The increased levels of RNA m6A modification and METTL3 expression were observed during the TGF-β-induced EMT in A549 and LC-2/ad lung cancer cells [84]. METTL3 knockdown reduced the m6A modification of JunB proto-oncogene-encoding mRNA (*JUNB*) and thus decreased its stability. Indeed, JunB is one of the most important transcription factors in the TGF-β-induced EMT of NSCLC cells [85]. Another vital EMT transcription factor, Snail, which represses E-cadherin expression and thereby contributes to cellular adhesion, was also found to be overexpressed by a mechanism mediated by METTL3. METTL3 was shown to deposit m6A modifications in the coding sequence of Snail-encoding mRNA, and as a result, promoted its YTHDF1-mediated Snail translation [86]. Notably, the m6A-dependent METTL3-mediated EMT was also reported in gastric cancer [87] and liver cancer cells [86]. Overall, these studies highlight the critical role of METTL3 expression in regulating the EMT-related oncogenes, the tumorigenicity of lung cancer cells, and in promoting lung cancer metastasis. m6A methylation of mRNAs as well as ncRNAs was also linked with the cellular metabolism in lung cancer. In a study by Jin et al. (2019), METTL3 increased m6A modification of Yes-associated protein (YAP)-encoding mRNA and *MALAT1* lncRNA, which induced resistance to cisplatin and metastasis in the NSCLC cells [88]. The level of translation of m6A-modified YAP-encoding mRNA increased as a result of recruitment of YTHDF1/3 and eIF3b to the translation initiation complex, whereas the methylation of *MALAT1* stabilized it and allowed it to sponge YAP-targeting miR-1914-3p, thus increasing the stability of YAP mRNA [88] (Figure 1A). Additionally, the precursor of miR-143-3p has been reported as a direct target of METTL3 in promoting brain metastasis of lung cancer. miR-143-3p targets 3′-UTR of vasohibin-1 (*VASH1*) mRNA and represses its expression, thereby promoting the EMT, invasion in an in vitro blood-brain barrier model, and angiogenesis of lung cancer cells [89]. This miR-143-3p/VASH1 axis has been shown to induce inversion of the NSCLC, thereby acting as a potential therapeutic target for cancer patients to improve their prognosis. 

#### 3.1.2. Involvement of m6A Readers in Lung Cancer

In addition to promoting tumorigenesis by the m6A writer mechanism, METTL3 was also shown to promote lung cancer while acting as an m6A reader. METTL3 can bind to the target m6A sites at 3-′UTR of mRNAs near the stop codon and interact with the eukaryotic translation initiation factor 3H (eIF3H) at the 5′ cap of the mRNA, thus promoting mRNA looping [79]. Furthermore, the METTL3-mediated translation regulates hundreds of target mRNAs associated with tumour progression and apoptosis (e.g., *EGFR*, *TAZ*, *MAPK2*, *DNMT3A*, and *BRD4*). The METTL3–eIF3h interaction is hence a potential therapeutic target for lung cancer patients.

The upregulation of an m6A reader, YTHDF2, was observed in lung cancer, which in turn promotes lung cancer by regulating the pentose phosphate (PPP) pathway. The study revealed that the PPP pathway supplies cancer cells with ribose 5-phosphate and NADPH and is regulated by the rate-limiting glucose-6-phosphate dehydrogenase (G6PD) enzyme. The binding between the m6A methylation site of 3′-UTR G6PD mRNA transcripts and YTHDF2 facilitates the translation of G6PD, thereby enhancing PPP flux [90] (Figure 1B). Another m6A reader, YTHDC2, also facilitates the growth of LUAD cancer cells by regulating the cystine/glutamate antiporter, system X_C_^−^, which facilitates glutathione (GSH) biosynthesis. System X_C_^−^ comprises two important components-solute carrier 7A11 (SLC7A11) and solute carrier 3A2 (SCL3A2). YTHDC2 binds to and promotes degradation of the m6A-containing *SLC7A11* mRNA [91] and homeobox A13 (HOXA13) mRNA [91], a transcription factor that induces SCL3A2 expression. Overall, the increased YTHDC2 reader protein expression and downregulation of both components of X_C_^−^ are important for the inhibition of tumour growth in LUAD [92].

#### 3.1.3. Involvement of m6A Erasers in Lung Cancer

The upregulation of FTO and ALKBH5 demethylases is significantly associated with their oncogenic roles in facilitating tumour cell proliferation and inhibiting apoptosis in LUSC. On the one hand, ALKBH5 was shown to act as an oncogene by causing demethylation of m6A sites in the *TIMP3* mRNA, thus decreasing its stability and consequent protein expression [93]. On the other hand, the downregulated tumour suppressor protein TIMP3 has also been implicated in prostate and gastric cancers and LUAD [93]. Additionally, ALKBH5 was demonstrated to contribute to LUAD progression by increasing the Forkhead box M1 (FOXM1) protein expression to induce hypoxia-mediated tumour proliferation [94]. The knockout of ALKBH5 in LUAD cells upregulated the level of m6A modification in *FOXM1* mRNA, thus reducing its association with polysomes and translation efficiency into FOXM1 protein [94]. A similar observation has also been reported for breast cancer cells [95].

On the other hand, FTO was demonstrated to promote lung cancer cell proliferation by removing m6A marks from ubiquitin-specific protease 7 (*USP7*) [96] and myeloid zinc finger 1 (*MZF1*) mRNAs [97] in NSCLC and LUSC subtypes, respectively. The demethylation of mRNA transcripts increased their stability and sequentially elevated their protein expression, thereby upregulating lung cancer cell growth. 

### 3.2. Involvement of m1A in Lung Cancer

**Figure 2:**
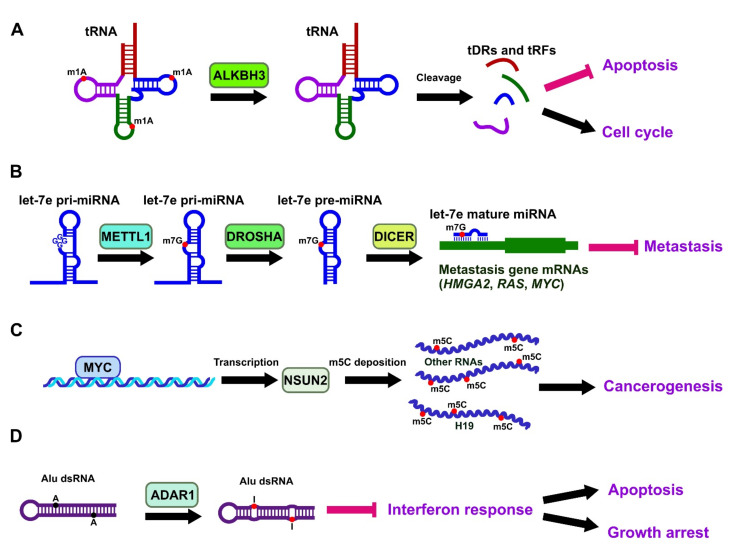
Some representative pathways of the involvement of RNA modifications other than m6A in lung cancer. (**A**) The removal of m1A marks from tRNAs by ALKBH3 leads to their processing into tDRs and tRFs which promote oncogenesis by inhibiting apoptosis and stimulating proliferation. (**B**) Deposition of m7A on let-7e pri-mRNA by METTL1 stimulates its processing into mature let-7e, which would suppress oncogenesis by inhibiting metastasis-associated mRNAs. (**C**) MYC transcription factor upregulates NSUN2, which increases total m5C methylation including H19 lncRNA associated with cancerogenesis. (**D**) ADAR1-mediated A-to-I editing of Alu-derived dsRNAs results in the inhibition of tumour suppressive interferon response, normally triggered by them.

The specific m1A demethylase ALKBH3, originally known as the prostate cancer antigen-1 (PCA-1), is highly abundant in prostate cancer [98,99]. Subsequently, the oncogenic role of ALKBH3 and m1A demethylation of mRNA has also been found in urothelial cancers [100], prostate cancer [99], breast cancer [101], colorectal [102], and NSCLC [103]. In NSCLC, the overexpression of ALKBH3 was found in both LUSC and LUAD. In particular, the percentage of ALKBH3-expressing cells in LUAD also correlated statistically with recurrence-free survival [103]. Furthermore, the knockdown of ALKBH3 in the LUAD cell line resulted in cell cycle arrest, senescence, and strong suppression of cell growth in vitro and reduced xenograft tumour growth in vivo [103]. In addition to mRNAs, m1A modifications were also found to occur in tRNAs. Indeed, it was shown in lung cancer cells that ALKBH3-mediated demethylation of tRNAs leads to an increased number of tRNA-derived small RNAs (tDRs) and fragments (tRFs) which can strengthen the ribosome assembly and increase the translation rate to prevent cell apoptosis [104] (Figure 2A). An NSCLC cell model showed that the tRF-Leu-CAG complex promoted G0/G1 cell cycle progression and subsequent cell proliferation concomitantly with the positive regulation of Aurora kinase A (AURKA) cell cycle regulator [74]. 

### 3.3. Involvement of m7G in Lung Cancer

METTL1 is a writer of m7G, the common 5′-cap modification of mRNAs and internal modifications in various noncoding RNAs. METTL1 was shown to methylate let-7e primary miRNA precursor (pri-miRNA) in lung cancer cells [64]. Such methylation disrupted the inhibitory secondary structure formed by a G-quadruplex within the pri-miRNA transcript, thereby allowing its further processing and production of the mature let-7e miRNA [64] (Figure 2B). The downregulation of mature let-7e would cause overexpression of its target genes, such as the high mobility group AT-hook 2 (*HMGA2*), *RAS* and *MYC* driver genes of metastasis, thus making METTL1-mediated m7G editing important for inhibiting lung cancer cell migration [64,105]. Similar findings have also been reported in colon cancer [106,107]. On the other hand, METTL1 overexpression was recently associated with poor prognosis and the downregulation of tumour suppressors in hepatocellular carcinoma, although the molecular mechanisms remain unknown [108].

### 3.4. Involvement of m5C in Lung Cancer

m5C is another epigenetic mark ubiquitously found on DNA, but has also been identified in rRNAs, tRNAs and mRNAs. m5C modification of RNA but not DNA was enriched in the circulating tumour cells from lung cancer patients, compared to whole blood cells [109]. Several lines of evidence indicate that RNA cytosine methyltransferases NSUN2 [110] and DNMT2 [111,112] are implicated in tumorigenesis, although their exact mechanisms remain unclear. The m5C writer NSUN2 is a direct target of MYC, a well-known regulator of tumour cell proliferation, which was first found upregulated in malignant skin tumours [113] (Figure 2C). Similar to MYC, NSUN2 was also highly expressed in various tumours [114,115]. Further studies on the potential roles of m5C in lung cancer development are required. NSUN2 may also exert its roles via lncRNAs. The upregulation of H19 lncRNA has been detected in many adult malignant lung cancer tumours. It was shown that NSUN2-mediated m5C modification of H19 resulted in its stabilization and pro-oncogenic effects [92]. 

### 3.5. Pseudouridine (Ψ, psi) in Lung Cancer 

Pseudouridine (Ψ, psi) regulates RNAs on a structural level by increasing their stability and altering their translational efficiency [116,117]. Ψ is the most abundant RNA modification generated by the Ψ synthase (PUS) enzymes (TruA, TruB, TruD, RsuA, RluA, and Pus10b). DKC1 is an RNA-dependent PUS whose binding with target RNA is guided by box H/ACA small nucleolar RNAs (snoRNAs). So far, mutations in the snoRNA-dependent Ψ synthase DKC1 have been associated with hereditary bone marrow failure syndrome, dyskeratosis congenita (DC), and consequent premature aging and cancer [118]. Downregulation of an H/ACA snoRNA, SNORA, which directs site-specific Ψ modification in the 18S rRNA subunit, resulted in the development of hepatocellular carcinoma in mice and humans [119]. Meanwhile, unlike in liver cancer, an elevated level of DKC1 and H/ACA snoRNAs and henceforth Ψ modification was found in the in vitro studies with prostate cancer cells [120]. Additionally, the role DKC1 Ψ writer plays in the transcriptional activation of pluripotency factors OCT4 and SOX2 has been shown in embryonic stem cells [119]. It remains to be seen how the level of DKC1 and other Ψ RNA modifications are associated with lung cancer cells, and their underlying mechanisms.

### 3.6. Adenosine-to-Inosine Editing in Lung Cancer

The role of editing enzymes in adenosine-to-inosine (A-to-I) modifications of mRNAs resulting in amino acid substitutions in proteins, and in tRNAs and miRNA-151, in cancer are well established [73,121,122]. Significant changes to the levels of A-to-I editing of Alu repetitive elements owing to the ADAR1 and ADAR2 enzymes expression were first identified in brain, oesophageal, breast, prostate, lung, kidney, and testis tumours in a transcriptome-wide RNA editing analysis [123,124,125]. However, their specific functions and mechanisms were later found to be highly dependent on the type of cancer. In LUAD tumours, RNA was shown to be over-edited, and ADAR was abundant at both the mRNA and protein levels [126]. ADAR enriched the expression of focal adhesion kinase (*FAK*) gene by binding to the *FAK* transcript and editing a specific intronic site that increased its mRNA stabilization. The overexpression of ADAR1 normally results in the inhibition of dsRNAs generated from transposable Alu repeats thus preventing them from triggering the interferon immune response [127]. The resultant shortage of dsRNAs and the suppression of the interferon immune response reduces apoptosis and growth arrest in the cancer cells. In other words, ADAR1 depletion can serve as a therapeutic approach in the immunotherapy of cancer patients by sensitizing cells to immune checkpoint blockades [128]. Another mechanism accounting for the oncogenic role of ADAR1 in lung cancer is the regulation of miRNA processing. Target transcripts such as miR-381 and *NEIL1* are tumour suppressors whose editing frequencies are enhanced by ADAR1 overexpression, resulting in significant growths of NSCLC cells [129]. The altered editing level of miRNAs demonstrates its potential as a biomarker of lung adenocarcinoma [130]. In a clinical setting, patients with ADAR1 amplification, despite having NSCLC diagnosed in the early stages, have poor outcomes, signifying the downstream effects of ADAR1-associated activation on the RNA editing patterns and prognosis [129]. 

The upregulation of ADAR1 in the prostate, liver, chronic myelogenous leukaemia (CML), colorectal and cervical cancers has been found to target cancer-related mRNAs such as *DHFR*, *BLCAP*, and *RRUNE1* [131]. Recently, Hu et al. demonstrated that RNA editing of the target mRNA encoding antizyme inhibitor 1 (AZIN1) could promote malignant NSCLC [40]. AZIN1 was first found in hepatocellular carcinoma (HCC) [132], and later in colorectal cancer. While promoting tumorigenesis, ADAR1 increased A-to-I conversion, and caused serine-to-glycine substitution in the AZIN1 protein [133]. This amino acid change in AZIN1 generates an isoform that strongly binds to the antizyme and inhibits antizyme mediated degradation of ornithine decarboxylase and cyclin D1. As a result, the high levels of ornithine decarboxylase and cyclin D1 increase polyamine uptake and henceforth cancer cell cycle progression, leading to increased OCT4 and SOX2 levels [134], cell proliferation, and tumour progression.

## 4. RNA Epigenetic Transcriptome Analysis in Lung Cancer

Until now there are still relatively few studies focusing on epitranscriptome-wide scale in lung cancer development. However, these studies really showed distinct differences and offered some clues for further research. For example, Li et al. [135] analysed The Cancer Genome Atlas (TCGA) database and found that most RNA methylation regulators showed distinct expression in tumour tissues and adjacent tissues, which may explain the statistical difference of five-year survival between high-risk group and low-risk group patients with LUAD [136]. In another study which analysed lung cancer data from multiple databases, it was reported that the expression levels of m6A-related proteins, such as HNRNPC, KIAA1429, RBM15, YTHDF1, YTHDF2, and METTL3 in cancer group were significantly upregulated, while FTO, METTL14, ZC3H13, YTHDC1 and WTAP were obviously downregulated. In this way, by enriching and analysing existing databases, gene risk signatures associated with RNA epigenetics can be developed for the diagnosis, treatment and prognosis of lung cancer.

## 5. Epitranscriptomic Modifications in Other Pulmonary Diseases

Besides regulating the progression of lung cancer, the role of m6A modifications in other pulmonary diseases such as chronic obstructive pulmonary disease (COPD) has also been reported. The occurrence of COPD has been associated with the expression of *IGF2BP3*, *ZNF217*, *METTL3*, *YTHDC1* and *YTHDC2* mRNAs. In a recent study, the differential expression of 24 common m6A RNA methylation regulators were bioinformatically analysed to identify interaction with the key COPD genes, using the STRING database [137]. The expression of *IGF2BP3* was upregulated in COPD while *FTO*, *ZNF217*, *METTL3*, *YTHDC1* and *YTHDC2* was downregulated compared with control samples. These m6A methylation regulators indirectly interacted with several key COPD-associated genes, such as *BCL2A1*, *GPX2*, *AKR1B10*, *ALDH3A1*, *CABYR*, *CYP4F3*, *EGF*, *UCHL1*, *CYP1A1*, *CYP1B1* and *MUCL1*. COPD is normally accompanied by the lethal disease pulmonary hypertension (PH), a progressive disease induced by postnatal chronic hypoxia [138]. Chronic hypoxia further triggers the over-proliferation of pulmonary artery endothelial cells (PAECs) and pulmonary artery smooth muscle cells (PASMCs), and the activation of quiescent fibroblasts. In PH-related genes, m6A modifications have also been shown to play a role. Recent studies suggest that the continuous low expression of METTL3 persists into adulthood to affect the m6A level in PH-related genes, resulting in long-term effects on lung development and pulmonary function. Using a postnatal hypoxia rat model, it was shown that the level of METTL3 was lower in the hypoxia group than in the control. In the HPH rat model, mRNA levels of *Tc1* and *Trps1* were increased in adult rats after postnatal hypoxia. In another study, hypoxia was shown to downregulate the expression of MEIS1 in pulmonary arteries which led to increased proliferation and migration of PASMCs [139]. This downregulation of MEIS1 was dependent on METTL14 m6A writer, which was highly upregulated under hypoxic conditions [139]. Additionally, PASMC proliferation can also occur via the STAT3 pathway [140]. This occurs when chronic normobaric hypoxia upregulates the expression of VGLL4, activating the SIRT1-mediated histone acetylation to contribute to pulmonary artery remodelling and the development of PH. Recently, the role of m6A modification was elucidated in a co-expression network of circRNA–miRNA–mRNA in HPH [141]. It was found in a rat model that hypoxia reduced the levels of m6A in circRNAs. In addition, two HPH-associated m6A-modified circRNAs, circXpo6 and circTmtc3, were found to mediate PH. Overall, these findings offer a new perspective to explain how m6A modifications participate in the pathogenesis of COPD, PH and lung development, shedding light on new directions of therapeutic development.

## 6. Future Perspectives and Conclusions

Dynamic and reversible, the role of RNA modifications in pathology has been established. The tumorigenic development of different types of cancer has been associated with different functions of RNA modifications. As epitranscriptomics-driven developments unfold, poor prognosis due to low treatment efficiency and failure of early diagnosis remain commonplace among cancer patients today. In lung cancer, the association of aberrant m6A, m1A, m5C, m7G, Ψ and A-to-I modifications with pathological process has been established. Together with the binding interactions between the target mRNA transcripts and modified nucleotides in the noncoding RNAs, such as miRNAs and circRNAs, RNA modifications participate in the genesis and progression of lung cancer by decreasing the stability or expression of mRNAs, encoding the suppressors of the oncogenes to reduce their inhibitory effects and enhancing the stability and expression of the pro-oncogenic transcripts. As a result, these regulatory proteins are of great potential to serve as novel diagnostic biomarkers and therapeutic targets for the early diagnosis and improved prognosis and treatment of lung cancer. 

However, understanding the exact mechanisms of these chemical modifications in both coding and non-coding RNAs remains incomplete and elusive. This review summarized the role of principal RNA modifications in lung cancer and other pulmonary diseases, but much remains to investigate the dysregulation of epitranscriptomic machinery implicated in the pathological processes. Further research may focus on the involvement of less investigated proteins, such as m6A-related VIRMA, RBM15, FMR1 and LRPPRC, and the effects of interactions between different RNA modifications, such as between m6A and m5C, on tumorigenesis. Furthermore, additional clinical trials are required to determine the potential diagnostic and therapeutic effects of RNA modifications among lung cancer patients.

## Figures and Tables

**Table 1:** Summary of six RNA modifications discussed in this review and their regulation machinery. Asterisks indicate the bona fide catalytic subunits.

Modification	Structure	Writer	Reader	Eraser
m6A	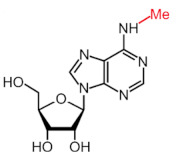	METTL3*, METTL16*; METTL14, WTAP, VIRMA, RBM15, RBM15B, ZC3H13	YTHDF1, YTHDF2, YTHDF3, YTHDC1, YTHDC2	FTO, ALKBH5
m1A	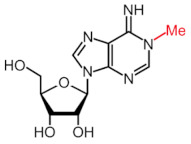	TRMT6, TRMT61A, TRMT10C, RRP8	YTHDF1, YTHDF2, YTHDF3, YTHDC1	FTO, ALKBH1, ALKBH3
m5C	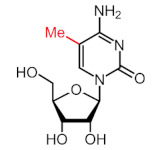	NSUN1-7, DNMT2	ALYREF, YBX1	TET2
m7G	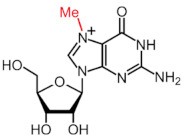	METTL1	-	-
Ψ	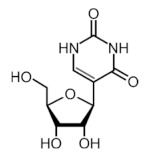	TRUB1, TRUB2, PUS1, PUS7	-	-
A-to-I	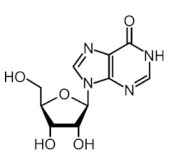	ADAR1, ADAR2	-	-

## Data Availability

All data presented this study are available from the corresponding author, upon responsible request.
